# Functional magnetic resonance imaging (fMRI) changes and saliva production associated with acupuncture at LI-2 acupuncture point: a randomized controlled study

**DOI:** 10.1186/1472-6882-8-37

**Published:** 2008-07-07

**Authors:** Gary Deng, Bob L Hou, Andrei I Holodny, Barrie R Cassileth

**Affiliations:** 1 Integrative Medicine Service, Memorial Sloan-Kettering Cancer Center, 1429 First Avenue, New York, NY 10021, USA; 2 Functional MRI Laboratory, Memorial Sloan-Kettering Cancer Center, 1429 First Avenue, New York, NY 10021, USA

## Abstract

**Background:**

Clinical studies suggest that acupuncture can stimulate saliva production and reduce xerostomia (dry mouth). We were interested in exploring the neuronal substrates involved in such responses.

**Methods:**

In a randomized, sham acupuncture controlled, subject blinded trial, twenty healthy volunteers received true and sham acupuncture in random order. Cortical regions that were activated or deactivated during the interventions were evaluated by functional magnetic resonance imaging (fMRI). Saliva production was also measured.

**Results:**

Unilateral manual acupuncture stimulation at LI-2, a point commonly used in clinical practice to treat xerostomia, was associated with bilateral activation of the insula and adjacent operculum. Sham acupuncture at an adjacent site induced neither activation nor deactivation. True acupuncture induced more saliva production than sham acupuncture.

**Conclusion:**

Acupuncture at LI-2 was associated with neuronal activations absent during sham acupuncture stimulation. Neuroimaging signal changes appear correlated to saliva production.

## Background

Acupuncture is a complementary and alternative medicine (CAM) modality that is practiced in many parts of the world for a variety of ailments[1]. It involves the insertion of fine needles at specified points on the skin. Most clinical research on acupuncture has focused on pain. Analgesic effects were reported in several trials [2-4]. Neuroimaging technologies have increasingly been used to explore the mechanism of action underlying acupuncture induced analgesia [5-10]. Questions have been raised on whether findings from analgesia models apply to other clinical settings where acupuncture has been practiced. In contradistinction to suppression of pain, acupuncture is sometimes used as a stimulus. Our interest has focused on saliva production: acupuncture has been shown to increase salivary flow in healthy volunteers [11], patients with Sjogren's syndrome[12] and those with radiation-induced salivary gland damage [12-14].

Using blood oxygen level dependent (BOLD) functional magnetic resonance imaging (fMRI) technology, we set out to investigate the neural substrates affected by acupuncture at a point (LI-2), an acupoint used in clinical practice [13,15-17] to treat xerostomia by stimulating saliva production. Here we report fMRI data related to acupuncture at this point in a randomized controlled trial of twenty healthy volunteers. The aim of this descriptive study was to explore what kind of fMRI changes may be associated with acupuncture stimulation at LI-2 and generate new leads for future research.

## Methods

### Study Subjects

Flyers describing this study were posted at our cancer center. Subjects who contacted us were evaluated according to the inclusion and exclusion criteria. Those eligible were enrolled in the study. Twenty subjects (10 males and 10 females) were enrolled after signing an informed consent form. The research protocol has been approved by the Memorial Sloan-Kettering Cancer Center Institutional Review Board. Inclusion criterion is: age 18 years and older (healthy volunteer). Exclusion criteria are: any major medical disorder requiring regular medical care; metallic implants making MRI contraindicated; inability to tolerate lying on MRI bed for the estimated 2 hours of study; sufficient knowledge of acupuncture allowing one to distinguish between the experimental and control interventions; inability to tolerate the placement of cotton gauzes in the mouth for measurement of salivation.

### Study design and interventions

This was a randomized, sham controlled, cross-over trial with each subject serving as his/her own control. Twenty healthy volunteers were subjected to both true acupuncture and sham acupuncture sessions, in random order (true acupuncture first, then sham acupuncture; or sham acupuncture first, then true acupuncture). Randomization was conducted by a secure database ensuring full allocation concealment. The subjects were blinded to group assignment. All subjects also received application of 1.2 ml lemon juice in the mouth in a separate session (data to be reported separately). True acupuncture was delivered at the LI-2 acupoint of the non-dominant hand. LI-2 is located on the radial side of the second digit in slight flexion, in the depression anterior to the metacarpophalangeal joint. Acupuncture at this point has been reported in the literature to help reduce xerostomia. Needles were manually manipulated by twisting after insertion. Sham acupuncture was provided by application of a sham (Streitberger) needle[18] at a non-acupoint on the ulnar side of the ipsilateral forearm, 3 cm lateral to the PC-6 acupuncture point. Sham needles, resting on top of the skin instead of penetrating the skin, were manually manipulated by twisting. BOLD fMRI images were obtained by applying a gradient echo pulse sequence and two boxcar paradigms (i.e., true and sham acupuncture paradigms). The true or sham acupuncture treatments were given one immediately after the other, with about 1 minute in-between.

### Instructions to the subjects

Subjects were told that they were participating in a study of neuroimaging changes associated with acupuncture and salivation because acupuncture was reported to help saliva production in patients whose salivary glands were damaged by radiation. They would receive two sets of acupuncture treatment. Cotton pads would be placed in their mouth then taken out to measure how much saliva they produce. After they came out of the MRI scanner, they were told that one of the acupuncture was supposed to be real and the other sham. They were then asked which acupuncture they thought they had received first.

### fMRI scans

A 1.5 Tesla (T) scanner (GE Signa equipped by Twinspeed hardware with a quadrature head coil) was used for the study. Plastic pads and tapes were used to immobilize the head within the head coil to minimize movement during the scans. High resolution T1-weighted images were obtained using a SPGR pulse sequence (TR/TE = 30 ms/14 ms, 90 degree flip angle, 256 × 256 matrix, 144–160 axial slices, 1.5 mm slice thickness with 0 cm gap for the whole brain coverage) and co-registered with the BOLD fMRI data which were acquired by using a T2* weighted EPI sequence(TR/TE = 5000 ms/40 ms, 90 degree flip angle, 128 KHz bandwidth, 128 × 128 matrix size, 45 axial slices, 4.5 mm slice thickness with 0 cm gap) and performing the true and sham acupuncture paradigms.

### fMRI paradigm for true acupuncture stimulation

**Figure 1:**
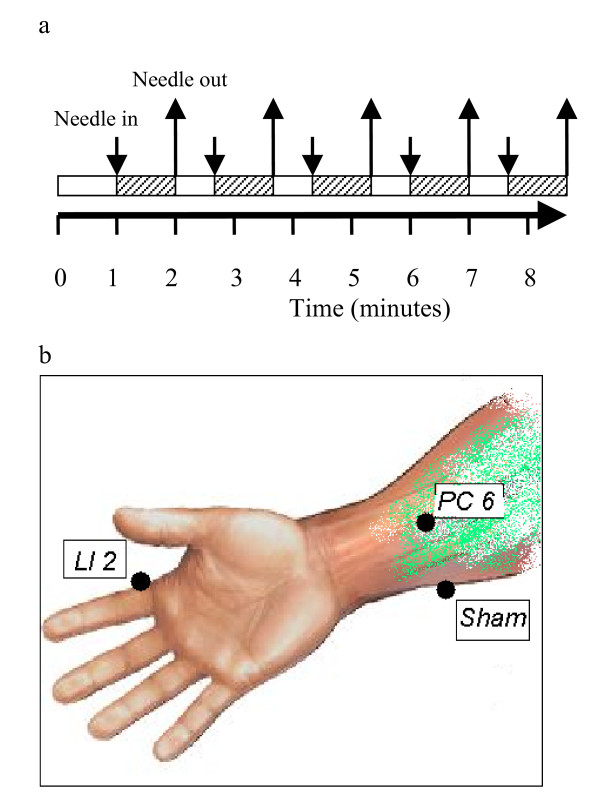
**Experimental Design**. Each subjects received both true and sham acupuncture in a randomized order. Boxcar design of fMRI paradigm was used. Each fMRI paradigm lasted a total of 8 minutes and 40 seconds. Stimulation (true or sham acupuncture) was initiated at the time points indicated with the downward arrows and stopped at those indicated with the upward arrows (panel 1a). During true acupuncture, a real acupuncture needle was inserted at the LI-2 acupuncture point. During sham acupuncture, a Streitberger placebo needle was applied at a non-acupuncture (sham) point (panel 1b).

A paradigm with a boxcar design was applied. Needles used for true acupuncture treatment were sterile disposable filiform stainless steel needles manufactured by Seirin Corporation, Japan. Size No.3 (0.20 mm) × 40 mm. The paradigm involved 5 stimulation periods (60 seconds) alternating with 5 resting (40 seconds) periods. The total duration was 8 min and 40 seconds (8'40"). Acupuncture was inserted at the LI-2 point of the non-dominant hand (on the radial side of the second digit in slight flexion, in the depression anterior to the metacarpophalangeal joint.) at time points 1'00", 2'40", 4'20", 6'00" and 7'40". The needle was removed at time points 2'00", 3'40", 5'20", 7'00" and 8'40" (Figure 1).

### fMRI paradigm for sham acupuncture stimulation

Needles used in sham acupuncture treatment are designed by K. Streitberger[18] and manufactured by Asia-Med, Germany. The needle has a blunt tip that does not penetrate the skin. Rather, it retracts into the handle when tapped and is supported vertical to the skin surface by a plastic ring and adhesive tape. Instead of insertion of a real acupuncture needle at LI-2 acupoint, the sham needle is applied to a point on the ulnar side of the ipsilateral forearm, 3 cm lateral to the PC-6 acupuncture point. PC-6 is located at 2 thumb-widths above the wrist crease between the tendons of palmaris longus and flexor carpi radialis. The timing of the stimulation and resting periods in the paradigm are identical to the timing in the true acupuncture stimulation (Figure 1).

### Evaluation of subject blindness

Upon completion of the session, subjects were asked whether they thought they received true or sham acupuncture first, and why. This was to account for effects from placebo effect.

### Measuring saliva production

Similar to as in [19], a pair of 2 inch by 2 inch sterile cotton gauzes was placed next to the parotid gland duct buccal opening, one on each side. The gauzes were pre-weighed. Subjects were instructed not to swallow during the period. The gauzes were removed after 8 minutes and 40 seconds and weighed again. The difference in weight represents the amount of saliva produced.

### Data Analysis

The functional MRI data acquired from the scanner were transferred to a LINUX work station and analyzed by using AFNI software (version: 2005_11_18_1920)[20]. The data processing includes motion correction. The motion curves (displacement in millimeter versus fMRI scanning time) were plotted. Seven subjects (6 females and one male) had head motion larger than 5 mm that was too great for accurately determining activation areas and were excluded from further analysis. One left handed subject was excluded from the final data analysis. Spatial smoothing with a 4 mm Gaussian filter was applied for blurring the data to increase the signal to noise ratio. All 26 data sets for the 13 included subjects and the two paradigms were performed in the individual and group analyses. A cross correlation coefficient (r) for the time course of each voxel and the input functions based on the paradigms' time patterns was calculated. The r value for the corresponding p value less than 0.05 was selected for the individual and group analyses to threshold the activation areas. A p value of less than 0.05 was considered statistically significant [21-23].

The group analyses were performed by applying an ANOVA algorithm with acupuncture (true or sham) as a fixed effect and subjects as the random effect. The activated areas for the individual analyses were colored and overlaid on the high resolution SPGR T1 weighted images. The activation areas showed in color in the images of the group analyses were overlaid to a standard Talairach brain. The coordinates for activation area (i.e., cortex) were determined by selecting the mid-point in the area for the slice with the most activation pixels, and were labeled based on the Talairach space.

Descriptive, comparative and correlative statistical analyses were performed using the build-in functions in Microsoft Excel.

## Results

The age of the subjects (ten male and ten female, all but one being right-handed) ranged from 22 to 58 years, with a median of 30. The order of needle stimulation was exactly balanced, with half of the sample randomized to receive true before sham acupuncture and half randomized to receive treatment in the reverse order. There was no evidence of unblinding: 8 patients guessed the order of treatments correctly, 5 incorrectly and 7 were unsure. There were no obvious differences in terms of the reasons given by subjects for their guess as to allocation.

### True acupuncture

**Figure 2:**
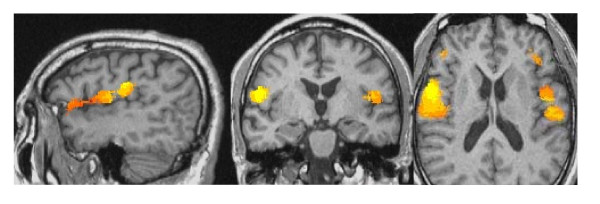
**fMRI changes associated with true acupuncture at LI-2**. Sagittal, coronal and axial view (respectively) of cortical areas activated by the true acupuncture paradigm.

The timing of acupuncture stimulation (true or sham) and the acupuncture points are shown in Figure 1. To evaluate changes induced by acupuncture, we conducted a group analysis (p < 0.05) in the twelve right-handed individuals who provided evaluable fMRI data. True acupuncture activated the parietal operculum, rolandic operculum, frontal operculum and insula (Figure 2). No regions of deactivation were observed. Talairach coordinates of the regions of interest (ROI) are shown in Table 1. Despite acupuncture being conducted on the left hand, bilateral activation was observed.

**Table 1:** Talaraich coordinates of regions activated by true acupuncture

Cortex	Left inferior frontal gyrus	Right inferior frontal gyrus	Left Pre Central Gyrus	Right Pre Central Gyrus	Left Insula	Right Insula	Left Middle Frontal Gyrus	Right Middle Frontal Gyrus	Left Post Central Gyrus	Right Post Central Gyrus
X(R-L)	-44(L)	44(R)	- 46(L)	55(R)	-46(L)	44(R)	-35(L)	44(R)	-60(L)	50(R)
Y(A-P)	25(A)	35(A)	6(A)	-3(P)	1(A)	-11(P)	40(A)	35(A)	-22(P)	-19(P)
Z(S-I)	4(S)	11(S)	8(S)	8(S)	12(S)	15(S)	9(S)	16(S)	26(S)	26(S)

### Sham acupuncture

**Figure 3:**
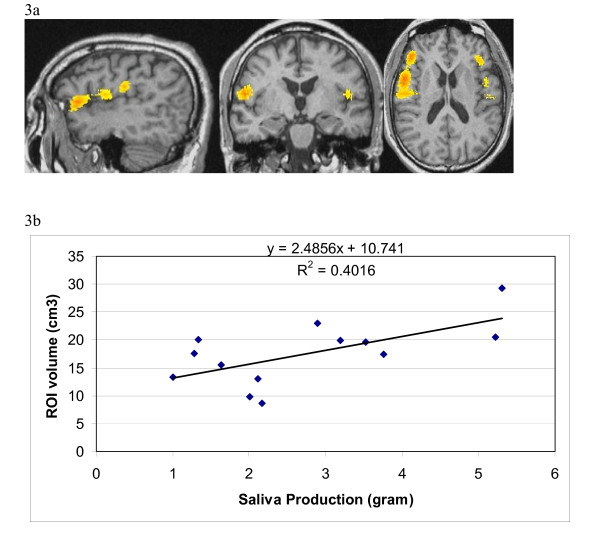
**Specific activation by true acupuncture versus placebo acupuncture**. Activation regions shown by fMRI signals associated with true acupuncture were compared to those from sham acupuncture. Panel 3a: sagittal, coronal and axial view (respectively) of activation in the insula and adjacent operculi. Panel 3b: Correlation of saliva production with ROI volumes.

To test against non-specific effects of cutaneous stimulation, we chose to use the sham (Streitberger) needle at a non-acupoint with the recipient blinded. This type of needle has a blunt end. Upon contact with the skin, it retracts into the handle without skin penetration. However, it does elicit a sensation similar to skin penetration. Its ability to blind the recipients has been validated[18]. A group analysis on sham acupuncture data demonstrated that the stimulation did not elicit activation in the above ROIs when compared to baseline. Nor did it elicit any other activation or deactivation detectable at this threshold (p < 0.05). When data from the true acupuncture group was compared directly to that from the sham acupuncture group, again the insular and adjacent operculum were activated (Figure 3a). In addition, activation was detected in the medial frontal gyrus (data not shown).

### Saliva production

True acupuncture led to a modestly but significantly higher amount of saliva production. Among the 13 subjects who provided evaluable fMRI data, average salivation in grams during the true and sham acupuncture phases was 2.72 (SD 1.42) and 2.38 (SD 1.43) respectively (two tailed paired t-test p = 0.02). Salivation was 0.34 g higher during the acupuncture phase than the sham phase (95% C.I. 0.10, 0.59). These data support previous clinical reports that acupuncture stimulates salivary flow [11-14]. Saliva production and insula/operculum ROI volume from the individuals have a Pearson correlation coefficient of 0.63. Linear regression of the two sets of values is shown in Figure 3b.

## Discussion

Acupuncture has been shown in clinical studies to have analgesic effects [2-4]. Several neuroimaging studies showed that acupuncture modulate activities in areas of the brain that are involved in pain signal processing. However, correlation between other physiological effects induced by acupuncture and the corresponding neuroimaging changes has not been well studied. Here we used a different experimental model, the acupuncture/xerostomia model, to investigate the possible mechanisms of action of acupuncture.

The pattern of fMRI activation we observed was interesting. By "pattern", we mean the totality of activation/deactivation areas. We observed some overlap with areas involved in pain perception. Pain can elicit activation of the sensorimotor cortices, rostral anterior cingulate cortex, insula, cerebellum, hippocampus, brain stem, etc. [9,24-29]. However, despite unilateral (on the left hand only) stimulation of subjects included in the final data analysis, we saw bilateral activation. We can not fully explain this finding by attributing it to painful stimulation alone.

The mechanisms of action of acupuncture in stimulating saliva production are unknown. It is possible that acupuncture at points in the head and neck area directly stimulates nerves innervating salivary glands. Another possibility is the placebo effect because expectation is well known to induce saliva production as in Pavlovian conditioning. A third possibility is that acupuncture acts by interacting with certain components of the neuronal network involved in salivation.

The neuronal matrix that controls salivation is not fully understood. Our current understanding indicates that it results from a complex interaction among many components of the peripheral and central nervous system, instead of a simple local reflex. Gustatory, olfactory and visual stimuli lead to activation of the insula, frontal operculum and rolandic operculum [30]. The insula and rolandic operculum receive a direct projection from the thalamic gustatory relay through bifurcate neurons[31]. Neuronal activity in this region is modulated by sensory input from taste receptors and lingual somatosensory receptors[30]. Pure gustatory stimuli and somato-gustatory stimuli both activate the insular lobe, the rolandic operculum, the frontal operculum and the temporal operculum[32]. Hypersalivation is observed during temporal lobe seizures [33]. During intraoperative mapping of a case of ictal hypersalivation, the seizures were identified as arising from the left anterior frontal operculum. After resection of epileptogenic opercular cortex, the seizures disappeared with no additional neurological deficits[34].

**Figure 4:**
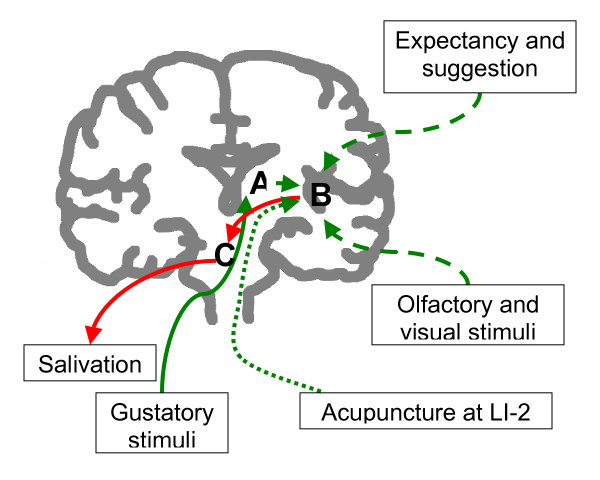
**Schematic representation of the gustation-salivation neuronal circuitry and how acupuncture may interact with it**. Green lines represent input neural signals, red lines output signals. Solid lines represent physiological response of salivation to gustatory stimuli. Dashed lines represent physiological signals from other cortices. Dotted lines represent the hypothetical pathway through which acupuncture activates the opercular and insular cortices. Areas A: thalamus; B: the insular and opercular cortices; C: salivatory nuclei in the pons.

Our data shows that areas activated by acupuncture at LI-2 overlap those involved in gustation/salivation[30]. But our study was descriptive in nature. We can not conclude that there is a causal relationship, i.e. such activation led to increased salivation. Nonetheless, our observations serve as a foundation for further hypothesis testing studies. A hypothesis we are proposing is illustrated in Figure 4. In this hypothesis, the insula and adjacent operculi are where gustatory, olfactory, visual stimuli and signals from expectation/suggestion are integrated. After the integration, signals are sent to the salivary nuclei in the pons which then go to the salivary glands via cranial nerves. Acupuncture, by activating the insula and adjacent operculi, taps into this circuit and produces the down-steam event of increased saliva production.

There are several limitations in our study. They were due to the exploratory nature of the study. Our original goal was to identify areas of interest, if any, that warrant further studies. It was not to test point specificity or other hypothesis. First, we included only one type of control intervention – sham acupuncture at a non-acupuncture point. We had considered using sham acupuncture at LI-2 or real needles at a non-acupuncture point as controls. We thought that the former might generate similar, although weaker, stimulation at the same location. We would then be comparing dose responses. In the latter case, we would be comparing point specificity which was not our original goal. To establish point specificity, we would have to compare true acupuncture at different acupoints and non-acupoints in one experiment, which would be a logical next step. Secondly, we did not ask the subjects to describe sensations, especially gustatory sensations, experienced during acupuncture. If acupuncture stimulates salivation via the gustatory cortices, one would expect to evoke such sensation. We also did not asked the subjects to describe "deqi" sensation, which is thought to be important for responses to acupuncture. A correlation between "deqi" sensation and fMRI changes would be an important finding.

## Conclusion

In this study, we show that acupuncture at a point commonly used to treat xerostomia is associated with activation of the insula and adjacent operculi. Such changes are not observed during sham acupuncture. True acupuncture also induces saliva production significantly more than sham. There is a positive correlation between the amount of saliva produced and changes in ROI volume. Our data suggest these areas are involved in the processing of stimuli at the LI-2 point by acupuncture. How the neuroimaging changes are linked to the physiological changes we observed deserve further investigation.

## Abbreviations

fMRI: functional magnetic resonance; BOLD: blood oxygen level dependent; CAM: complementary and alternative medicine; ROI: region of interest.

## Competing interests

The authors declare that they have no competing interests.

## Authors' contributions

GD conceived the study, participated in its design and coordination and drafted the manuscript. BLH participated in study design, carried out fMRI data acquisitions and analysis, and edited the manuscript. AIH participated in study design, data interpretation and manuscript preparation. BRC participated in study design and manuscript preparation. All authors read and approved the final manuscript.

## Pre-publication history

The pre-publication history for this paper can be accessed here:


